# Factors Influencing the Waste Separation Behaviors of Urban Residents in Shaanxi Province during the 14th National Games of China

**DOI:** 10.3390/ijerph19074191

**Published:** 2022-04-01

**Authors:** Daoyan Guo, Xinping Wang, Taozhu Feng, Shuai Han

**Affiliations:** 1 School of Management, Xi’an University of Science and Technology, Xi’an 710054, China; fengtz@xust.edu.cn; 2 School of Management, Fudan University, Shanghai 200433, China; 3 Energy Economy and Management Research Center, Xi’an University of Science and Technology, Xi’an 710054, China; 4 College of Economics and Management, Shandong University of Science and Technology, Qingdao 266590, China; hessa1222@163.com

**Keywords:** China, municipal solid waste, waste separation behavior, urban residents, policy suggestion

## Abstract

The behaviors of urban residents in terms of waste separation at source are crucial to achieve the reduction and recycling of waste in urban governance. In this study, the data were collected from 1008 respondents in Shaanxi province, the host of China’s 14th National Games; theoretical and empirical analyses were conducted to determine the factors influencing waste separation behaviors, and specific policy suggestions are proposed. The results show that the waste separation behaviors of urban residents are positively influenced by their cognition of waste pollution, ecological values, sense of social responsibility, perceived knowledge, and perceived utilities of waste separation. Moreover, the residents’ waste separation intentions partially mediate the relationships between their cognition of waste pollution, ecological values, perceived utilities, and waste separation behaviors, which almost totally mediate the relationship between their sense of social responsibility and waste separation behaviors. In addition, it was shown that the cost of waste separation can weaken the effect of waste separation intention on behavior, while waste separation facilities, social norms, and the publicity for China’s 14th National Games have the opposite effect. Finally, policy suggestions are proposed to encourage urban residents to adopt waste separation behaviors, contributing to transforming Shaanxi into a zero-waste province.

## 1. Introduction

Municipal solid waste (MSW) causes adverse impacts on urban environments, but it is also an important resource that can be recycled [1,2]. In order to turn waste into wealth and solve the increasing contradiction between the supply and demand of resources, it is essential to reduce the volume of garbage and make a collection of reusable and recyclable materials [3]. The Chinese government is determined to further promote waste separation to contribute to building a beautiful China. According to statistics obtained from the Ministry of Ecology and Environment, 196 large- and medium-sized cities in China generated 235.602 million tons of MSW in 2020, and Shaanxi province was the 10th highest garbage-producing region in the country. In addition, the 14th National Games of China will be held in Shaanxi in September 2021. Therefore, the effective management of MSW in Shaanxi is not only an important task for preserving and improving the ecological environment, but it is also a necessary requirement to create a good image of the province.

The provincial government in Shaanxi considers the source separation of MSW as very important. By the end of 2021, nine demonstration zones at the provincial level had been set up in Shaanxi province, namely the new districts of Qujiang and Tongchuan and the districts of Chang’an, Qindu, Jingkai, Beilin, Lianhu, Jintai, and Linwei. In these areas, more facilities are available, for instance, sorting centers for recyclables, temporary storage containers for hazardous waste, and vehicles for transporting various types of MSW. In addition, the government has released a series of laws and regulations, such as the “Implementation Plan for the Domestic Waste Classification System in Shaanxi Province” and the “Construction Standards on the Demonstration Zones of Domestic Waste Classification in Shaanxi Province (on trial)”. However, a large number of residents do not separate MSW, which results in a poor performance of waste separation policies [4,5]. There is ample evidence of widespread mixed transportation and disposal of MSW because waste is not always recycled in the right container. It is noteworthy that the current standards, plans, and regulatory policies concerning waste separation were established by taking the government’s perspective. In contrast, the standpoint of participants in terms of the feasibility and effectiveness of related policies was neglected, thereby causing low participation to become a prominent problem in the promotion of waste separation. In fact, if the participants’ attitudes are not considered in the design and application of waste separation policies, and inappropriate policies are adopted to restrict their actions, a behavioral rebound is likely to be triggered after deregulation [6], and this goes against the trend of multi-stakeholder involvement in environmental governance [7]. Hence, the encouragement of urban residents to spontaneously participate in waste separation is an important aspect for the achievement of MSW source separation in Shaanxi province.

Studies have shown that the waste separation behaviors of urban residents are affected by individual factors [8,9,10] and external factors, such as the presence of separation facilities and social norms [11,12,13]. In fact, human behavior is jointly determined by multiple factors. Urban residents always carefully consider their decision of sorting domestic waste into different types of bins, but numerous studies have only analyzed the effects of single factors on waste separation behaviors. In addition, urban residents in Shaanxi province are likely to take positive actions to contribute to building a good image of the region, as it is the location of the 14th National Games of China. Thus, it is intriguing and worth exploring whether the publicity for this event has positive effects on their waste separation behaviors.

Based on the above analysis, this study aims to identify the factors that influence the waste separation behaviors of urban residents in Shaanxi province and clarify their multiple effects, thereby proposing policy suggestions to encourage urban residents to adopt such behaviors. This study contributes to enriching environmental behavior theories by systematically demonstrating the influencing mechanisms of multiple factors on MSW separation behaviors during the 14th National Games of China. The proposed policy suggestions provide practical guidance to solve the problem of MSW source separation.

## 2. Theoretical Analysis and Hypothesis Design

### 2.1. Direct Influences of Waste Separation Awareness on Behavior

Behaviors can be explained and predicted by their corresponding types of awareness based on the theories of planned behavior, habitual behavior, and knowledge-attitude-practice [14,15,16]. Behaviors arise from the activation of awareness, and residents seldom take action when holding negative perceptions, beliefs, and values. In this study, the responses obtained from open-ended interviews showed that waste separation awareness is the predisposing factor of ensuring behaviors, and it includes the cognition of waste pollution, ecological value, sense of social responsibility, and perceived knowledge and utility of waste separation.

Residents’ cognition of waste pollution measures their knowledge and understanding of the harmful effects of domestic waste pollution and the urgency of solving pollution problems. Numerous theories on human behavior indicate that individual cognition is the internal premise of behavioral intentions and ensuing actions [15,16,17,18]. Moreover, some studies have empirically analyzed the relationship between individual cognitions and environmental behaviors. For example, pro-environmental behaviors, such as low-carbon and ecologically responsible behaviors, could be positively affected by the public cognition of climate change [19,20,21,22]. Therefore, urban residents are likely to adopt waste separation behaviors when they have high levels of cognition of waste pollution.

Value is the basic criterion of concrete behaviors, and these behaviors also reflect the value orientations of residents. The value-belief-norm theory, value-attitude-behavior theory, and precede-proceed model suggest that individual value decisively influences behaviors and behavioral intentions and that the ecological value is closely related to environmental behaviors [15,23,24]. Empirical evidence showed that pro-environmental behaviors are positively influenced by the individual ecological value [25,26]. For instance, an investigation based on 1892 urban residents in eastern China indicated that their approach-avoidance responses to personal carbon trading scheme were affected by the ecological value, and residents holding this ecological value were more likely to accept the scheme [27]. The implication of this is that residents are likely to take waste separation actions when they hold high levels of ecological value.

According to the theory of responsible environmental behavior proposed by Hines et al. (1987) [28], the sense of social responsibility is a critical factor for the determination of residents’ pro-environmental behaviors [26,29]. Researchers pointed out that residents holding a higher sense of social responsibility were more likely to adopt ecological behaviors, which supports the theory of responsible environmental behavior. For example, an empirical study indicated that people directly burned their waste when they had a lower sense of social responsibility [30]. Yang et al. (2016) [31] found that there was a greater likelihood of taking energy curtailment actions if Chinese urban residents had a higher sense of environmental responsibility, and Yue et al. (2020) [32] demonstrated that this responsibility had a positive influence on green consumption behaviors. In addition, Guo et al. (2021) [33] showed that the sense of social responsibility of Chinese urban residents could promote their willingness to accept a personal carbon trading scheme. Therefore, it can be concluded that MSW source separation behaviors probably stem from the activation of an individual sense of social responsibility, and if it is high, residents are much more likely to participate in waste separation activities.

The perceived knowledge of environmental conservation is the internal premise of forming environmental behaviors based on a number of typical models, such as the knowledge-belief-practice model, the precede-proceed model, and the environmental literacy model [15,16,18,34]. For example, Rausch and Kopplin (2021) [35] demonstrated the positive influence of consumers’ environmental knowledge on their purchase intentions in relation to sustainable clothing. Guo et al. (2021) [27] found that urban residents were usually unwilling to participate in carbon trading when they barely understood what personal carbon-trading schemes are. Thus, it can be assumed that the residents’ perceived knowledge of waste separation is the prerequisite for adopting waste separation behaviors.

Based on theories of planned and interpersonal behavior, the perception of positive or negative results derived from specific behaviors can further influence individual environmental behaviors [14,36]. Some studies maintain that the perception of positive behavioral effects could contribute to the implementation of pro-environmental behaviors. For example, Wei et al. (2018) [37] found that the improvement of carbon capability of urban residents depended on their perception of utility experience. In this study, the perceived utility of waste separation measures the individual perception of the significance, efficiency, and effect of MSW separation, which endows residents with psychological rights and affects their inner beliefs, thus significantly influencing their practical waste separation actions.

Based on the above analysis, the following hypotheses were proposed:

**H1a:**  *The urban residents’ cognition of waste pollution* *can positively influence their waste separation behaviors.*

**H1b:**  *The urban residents’ ecological* *values can positively influence their waste separation behaviors.*

**H1c:**  *The urban residents’ sense of social responsibility* *can positively influence their waste separation behaviors.*

**H1d:**  *The urban residents’ perceived knowledge of waste separation can* *positively influence their waste separation behaviors.*

**H1e:**  *The urban residents’ perceived utility of waste separation can* *positively influence their waste separation behaviors.*

### 2.2. Mediating Influences of Waste Separation Intention on the Relationship between Awareness and Behavior

According to mainstream behavioral theories (e.g., the theories of reasoned action, planned behavior, and interpersonal behavior), behavioral intention is the direct determinant of behavior, and through it, all factors indirectly affect behavior [14,23,36]. Empirical evidence showed that people were more likely to take action when they had stronger behavioral intentions. For instance, an investigation of 265 wholesalers in Kermanshah, western Iran, reported that behavioral intention had a powerful and positive impact on the behavior adopted to manage fruit and vegetable waste [38]. In this study, waste separation intention indicates the behavioral tendency of urban residents to take MSW separation actions, and it reflects the effort they are willing to make for it. The responses obtained from the open-ended interview showed that there was usually a higher possibility of voluntary participation in waste separation when the residents’ intentions were stronger. Therefore, the intention to separate waste probably plays a mediating role in the relationship between waste separation awareness and behavior, and thus, the following hypotheses were made:

**H2a:**  *The urban residents’ waste separation intentions can mediate the* *relationship between their cognition of waste pollution and behaviors.*

**H2b:**  *The urban residents’ waste separation intentions can mediate the* *relationship between their ecological values and behaviors.*

**H2c:**  *The urban residents’ waste separation intentions can mediate* *the relationship between their sense of social responsibility and behaviors.*

**H2d:**  *The urban residents’ waste separation intentions can mediate* *the relationship between their perceived knowledge of waste separation and behaviors.*

**H2e:**  *The urban residents’ waste separation intentions can mediate* *the relationship between their perceived utility of waste separation and behaviors.*

### 2.3. Moderating Influences of Situational Factors on the Relationship between Waste Separation Intention and Behavior

According to the attitude-behavior-context theory, environmental behavior is determined by the interaction of inner attitudes and situational factors, and the latter can moderate the relationship between individual attitudes and environmental behaviors [24,39]. These factors commonly include economy, culture, technology, and surroundings. In this study, the situational factors of waste separation behaviors were identified as the cost of waste separation, waste separation facilities, social norms, and the publicity for China’s 14th National Games.

The cost of waste separation is an enabling factor that turns intention into behavior. In general, individuals tend to achieve their goals at the minimum cost, which comprises both monetary and non-monetary costs. On one hand, when an individual adopts source separation behaviors, the monetary cost is largely generated by the essential purchase of garbage bags and cans. It is highly likely that individuals will not voluntarily take separation actions in the long term if MSW separation requires a high monetary cost. Consequently, the monetary cost of waste separation will moderate the influence of waste separation intention on behavior. On the other hand, the non-monetary cost refers to the residents’ physical and mental costs derived mainly from the acquisition of knowledge on waste separation and development of waste separation habits. The MSW separation policies deployed in Shaanxi province establish that urban residents should familiarize with the required separation standards and then begin to regularly separate and correctly throw away their waste. In fact, individual habits are usually formed by repeated practice, and they greatly influence the modification of current behaviors [15,36]. For instance, De Vries et al. (2011) [40] found that the habit of switching the lights off upon leaving a room could facilitate the transformation of intention into practical actions (switching lights off). Hence, non-monetary costs are likely to moderate the adoption of waste separation behaviors. Based on the above analysis, the following hypothesis was proposed:

**H3a:**  *The cost of waste separation can moderate the relationship between the* *waste separation intentions of urban residents and their behaviors.*

Source separation facilities represent a reinforcing factor for the adoption of waste separation behavior. According to the precede-proceed theory, the availability of various technical resources is a premise for adopting certain behaviors [15,41]. Empirical research has shown that pro-environmental behaviors, such as waste separation and green consumption, could be affected by product maturity and convenience of infrastructure [37,42]. In practical terms, the fact that many urban residents in Shaanxi province do not take action can be attributed to shortage of MSW separation facilities or to the fact that trash containers are not labeled in accordance with waste-segregation objectives. It is difficult to translate a strong intention into practical action when the waste separation facilities cannot satisfy the conditions for the adoption of behaviors. Hence, waste separation facilities are likely to play a moderating role in the relationship between waste separation intention and behavior, and the following hypothesis was made:

**H3b:**  *Waste separation facilities can moderate the relationship between the waste* *separation intentions of urban residents and their behaviors.*

Social norms, which are regarded as shared standards of acceptable behaviors by groups, can strengthen or weaken individual intention-behavior relationships [15,41]. In this study, social norms are considered to influence waste separation behavior in regard to three aspects. First, the attitudes and behaviors of reference groups will be regarded as important information when residents do not have a sufficient knowledge of waste separation. In other words, social norms have informational social effects on the waste separation behavior of residents. Second, residents usually conform to the dominant norms of social groups in order to gain recognition, increase respect, or avoid alienation, and thus, social norms probably exert normative social impacts on waste separation behaviors. For example, Deniz (2013) [43] demonstrated that individuals were afraid of feeling alienated from others, and they were also under pressure from reference groups, thereby following or disregarding certain behaviors. Third, in keeping with the behaviors of reference groups, residents consciously accepted, followed, and internalized their attitudes and actions. It is quite possible that social norms can have value-expressive influences on the waste separation behaviors of urban residents. In summary, as social norms are likely to cause moderating effects on the relationship between waste separation intentions and behaviors, the following hypothesis was made:

**H3c:**  *Social norms can moderate the relationship between* *the waste separation intentions of urban residents and their behaviors.*

China’s National Games, also known as the country’s “mini-Olympics”, always attract considerable attention. Notably, the 14th National Games are scheduled to be held in Shaanxi province. According to the attitude-behavior-context theory, the publicity for the games may represent a specific situational factor that can influence the daily behaviors of urban residents in the region [24,39]. In particular, the publicity for the games may modify their waste separation decisions by enhancing or reducing the dependence of their behaviors on behavioral intentions. For instance, many residents in Shaanxi province may decide to adopt separation behaviors after watching the games’ theme (i.e., “games for all, together in mind and action”) even though they have weak intentions to take MSW separation actions. Therefore, the publicity for the games is likely to exert a weaken or strengthen the residents’ waste separation intentions on consequent behaviors, and the following hypothesis was made:

**H3d:**  *The publicity for China’s 14th National Games can moderate the* *relationship between the waste separation intentions of urban residents and their behaviors.*

## 3. Materials and Methods

Based on qualitative analysis, the following variables were determined: waste separation behavior and intention, cognition of waste pollution, ecological value, sense of social responsibility, perceived knowledge and utility of waste separation, cost of waste separation, waste separation facilities, social norms, and publicity for China’s 14th National Games. As a questionnaire prerequisite, scale development has a serious impact on the reliability of the research results. Some initial questionnaire items were derived from existing scales, while others were creatively designed based on respective conceptions of different variables, residents’ responses to interview questions, and expert advice. For example, three items were included to evaluate the publicity for China’s 14th National Games factor, namely: “I know that Shaanxi province will host the 14th National Games in September 2021”, “Public service advertisements about China’s 14th National Games can be seen everywhere in stations, supermarkets, schools and other public places”, and “I feel a strong atmosphere created by China’s 14th National Games in Shaanxi province”. In addition, all measurement items were rated on a five-point Likert scale, and respondents were required to make judgments based on their actual situations and real thoughts. Preliminary investigations were conducted through the Wenjuanxing online survey website from 1 to 10 August 2021, and 209 valid questionnaires were collected. Measurement scales were quantitatively examined using IBM SPSS Statistics v22.0 (New York, United States), and they were also evaluated by five experts in the field of environmental behavior and nine urban residents. Moreover, all scales were proven to have high reliability and validity, and thus, they could be used for conducting formal investigations.

The present study specifically focused on urban residents in Shaanxi province, and these were selected as respondents. The formal investigations were conducted between 10 and 30 September 2021. In order to improve data validity, four measures were taken during the investigation process. First, a commitment to data security (i.e., the information collected would be only used for scientific purposes) was included on the preface of every questionnaire copy. Second, monetary payoffs were provided to increase the enthusiasm and involvement of residents. Third, structured one-on-one interviews were conducted for some elderly respondents that needed assistance to fill in the questionnaire. Finally, a filtering process was adopted to eliminate invalid data. If one answer was missing, or eight consecutive answers were the same, the questionnaire was considered invalid. In summary, a total of 1200 questionnaires were collected from urban residents in Shaanxi province, and of these, 1008 were the valid.

Table 1 reports the demographic distribution of the 1008 respondents in Shaanxi province. The female and male respondents were 502 and 506, respectively. In terms of age, respondents aged 31–40 years old accounted for 20.14% of the total, which was the highest proportion. The respondents holding a master’s or doctorate degree accounted for only 1.49% of the total, and the largest proportion of respondents (34.72%) had only received a primary or middle school education. With respect to annual income, respondents with less than CNY 30,000 of annual individual income were the largest group (33.43%), while those with CNY 100,000–200,000 of annual household income made up the largest proportion of the total (47.62%). In addition, respondents were distributed by the place of residence as follows: 235 respondents resided in Xi’an, 81 in Xianyang, 80 in Weinan, 92 in Baoji, 76 in Tongchuan, 72 in Yan’an, 75 in Yulin, 83 in Hanzhong, 82 in Ankang, 72 in Shangluo, and 60 in Yangling Demonstration Zone. Overall, the demographic distribution of the 1008 respondents was representative of the reality of Shaanxi province.

## 4. Results

### 4.1. Descriptive Statistics of Waste Separation Behavior and Its Driving Factors

The descriptive statistics of main variables are shown in Table 2. The mean value for waste separation behavior was 3.67, implying that a considerable number of urban residents in Shaanxi province took waste separation actions. Obviously, the mean value of waste separation intention (4.00) was greater than that of waste separation behavior. It was unexpected that some residents did not adopt separation behaviors even though they had strong behavioral intentions to do it. In regard to waste separation awareness, the average score for perceived knowledge of waste separation reached 3.81, indicating that most of the urban residents knew how to carry out the separation of MSW at source. It should be noted that a strong atmosphere was created in Shaanxi province, as the location of China’s 14th National Games, because the mean score for this variable reached 4.01. In addition, the mean of waste separation cost recorded a small value of 2.95; therefore, it was inferred that urban residents did not consider MSW separation particularly expensive or troublesome.

### 4.2. Empirical Analysis of the Influence of Waste Separation Awareness on Behavior

Multiple linear regression was adopted to test the effects of the residents’ waste separation awareness on their behavior. The results are shown in Table 3. The model was significant at the 0.01 level, and the adjusted R^2^ reached 0.709; thus, the model fitted the data well. Moreover, waste separation behavior was positively correlated with the five examined factors at the 0.01 level of significance, which supported hypotheses H1a to H1e. To be more specific, the waste separation behaviors of urban residents can be directly and positively driven by the cognition of waste pollution, ecological value, sense of social responsibility, perceived knowledge of waste separation, and perceived utility of waste separation.

### 4.3. Empirical Analysis of the Mediating Influences of Waste Separation Intention on the Relationships between Awareness and Behavior

The following three steps were taken to test the mediating effects of waste separation intention. First, the coefficients (*c_1_*) of waste separation awareness were measured using the regression model of the effects of waste separation awareness on behavior. It was established that there were no mediating effects if *c_1_* was not significant. Second, the coefficients (*a*) of waste separation awareness were estimated in the regression model of the effects of waste separation awareness on intention. Third, the coefficients of waste separation awareness (*c_2_*) and waste separation intention (*b*) were evaluated in the regression model of waste separation awareness, intention, and behavior. Table 4 shows the results of the test on the mediating effect of waste separation intention. In the path “SSR → WSI → WSB”, both *a* and *b* were significant, while *c_2_* was not significant. Thus, the relationship between sense of social responsibility and waste separation behavior was totally mediated by waste separation intention, which supported hypothesis H2c. It was notable that *a* was not significant, and *b* was significant in the path “PKWS → WSI → WSB”; therefore, the Sobel test was adopted for the identification of mediating effects [44]. The results showed that hypothesis H2d was not supported, and thus, waste separation intention had no mediating effect on the relationship between perceived knowledge of waste separation and waste separation behavior. In addition, all coefficients were significant at the 1% level in the other paths (“CWP → WSI → WSB”, “EV → WSI → WSB”, “PUWS → WSI → WSB”), which indicated that the relationships between cognition of waste pollution, ecological value, perceived utility of waste separation, and waste separation behavior could be partially mediated by waste separation intention. Hence, hypotheses H2a, b, and e could be supported. The proportion of mediating influence to the total effect was also estimated in order to report the direct and mediating effects. For instance, in the path “CWP → WSI → WSB”, the direct effect was 0.135 (*c_2_* = 0.135), the mediating effect was calculated as 0.07371 (*ab* = 0.315 × 0.234 = 0.07371), and the total effect was 0.209 (*c_1_* = 0.209); thus, the proportion of mediating effect could be expressed as 35.27% (0.07371/0.209 × 100% = 35.27%).

### 4.4. Empirical Analysis of the Moderating Influences of Situational Factors on the Relationship between Waste Separation Intention and Behavior

Hierarchical regression was used to test the moderating influences of situational factors. Table 5 shows the results of the test on the moderating effect of waste separation cost. The adjusted R^2^ of models 1, 2, and 3 were 0.566, 0.572, and 0.580, respectively, indicating that the fit of model 3 was better than that of models 1 and 2. Moreover, the coefficient for interaction term (−0.137) was significant at the 0.01 level. Therefore, the cost of waste separation could significantly moderate the relationship between the waste separation intentions of residents and their behaviors, which supported hypothesis H3a. Specifically, the cost of waste separation weakened the effect of waste separation intention on behavior.

Table 6 shows the results of the test on the moderating effect of waste separation facilities. It is clear that model 3, whose adjusted R^2^ was larger than that of models 1 and 2, had a better fit. Furthermore, the coefficient for interaction term (0.101) was significant at the 0.01 level. Hence, the waste separation facilities factor strengthened the effect of waste separation intention on behavior. In summary, waste separation facilities had a moderating effect on the relationship between the waste separation intentions of residents and their behaviors, which supported hypothesis H3b.

Table 7 presents the results of the test on the moderating effect of social norms. Model 3, whose adjusted R^2^ exceeded that of models 1 and 2, had the best fit. Further analysis showed that the coefficient for interaction term was 0.072, and it was significant at the 0.01 level. Consequently, social norms played a moderating role in the relationship between the waste separation intentions of residents and their behaviors, which supported hypothesis H3c. To be more precise, social norms strengthened the effect of waste separation intention on behavior.

Table 8 shows the results of the test on the moderating effect of the publicity for China’s 14th National Games. The adjusted R^2^ of models 1, 2, and 3 were 0.566, 0.568, and 0.594, respectively, showing that the latter was the best. The coefficient for interaction term (0.266) was significant at the 0.01 level, indicating that the publicity for China’s 14th National Games strengthened the effect of waste separation intention on behavior. Therefore, this factor had a moderating effect on the relationship between the waste separation intentions of residents and their behaviors, which supported hypothesis H3d.

## 5. Discussion

The mean values of waste separation intention and behavior were 4.00 and 3.67, respectively, which indicated an obvious gap between the two factors. In practical terms, it was shown that many urban residents of Shaanxi province do not adopt separation behaviors even though they hold strong behavioral intentions in regard to this issue. The fact that people just say “yes” instead of taking action has been identified as a prevalent phenomenon. For example, Echegaray and Hansstein (2017) [45] found that most consumers in Brazil had positive intentions toward recycling electronic appliances, but only a few of them took actual actions in this regard. Similarly, Rausch and Kopplin (2021) [35] demonstrated the intention-behavior gap in regard to the purchase of sustainable clothing. In fact, this gap results from people being inevitably affected by the external environment, which includes regional culture and social norms [46,47]. Although this is a common phenomenon, there is still a need to convert the residents’ waste separation intentions into spontaneous behaviors to better achieve the zero-waste goal. Therefore, it is necessary to identify the crucial factors affecting the intention-behavior gap in relation to MSW separation in Shaanxi province and provide policy suggestions for bridging such gap.

In Shaanxi province, the host of China’s 14th National Games, extensive publicity has been undertaken revolving around the theme “games for all, together in mind and action”. This publicity was demonstrated to strengthen the relationship between waste separation intention and behavior, which is an intriguing fact and one that is worth further exploring. The possible reason for this is that place identification plays a role in the moderating effect. The strong atmosphere created in Shaanxi province because of the hosting of China’s 14th National Games is likely to increase the sense of place identification and attachment of local residents. Moreover, empirical research showed that place identification could exert an indirect influence on environmental behaviors. For example, Vaske and Kobrin (2001) [48] indicated that it had a mediating effect on the relationship between place dependence and environmentally responsible behavior. In fact, as a substructure of social identity, place identification represents the emotional bond between a person and a place. When people become identified with a certain place, they generally adopt more environmentally friendly behaviors in their daily activities. Therefore, the residents’ enhanced sense of place identification is enhanced by the publicity for China’s 14th National Games, thereby strengthening the relationship between waste separation intention and behavior.

## 6. Policy Suggestions

Based on the current state of MSW separation behaviors of urban residents in Shaanxi province and the factors that influence such behaviors, the following policy suggestions are proposed to encourage urban residents toward the adoption of waste separation behaviors.

The government should make it a top priority to increase the waste separation awareness of all urban residents. On one hand, education, propaganda, and MSW separation services should be promoted in schools in order to drive students to convert waste separation awareness into voluntary behaviors, thereby encouraging the new generations to develop the good habit of waste separation. On the other hand, it is necessary to establish a social service system dedicated to MSW separation to improve the waste separation awareness of all residents as well as their behaviors. For example, initiatives could include the extensive dissemination of knowledge about the issue in every community through practical activities, using a variety of posters, quizzes, comic dialogues, etc.

Creating a positive atmosphere around the issue of waste separation would lead to an increase of the residents’ involvement. The importance, standards, facilities, measures, and effects of MSW separation should be comprehensively and objectively reported by both traditional and new media platforms (e.g., Weibo, WeChat, Toutiao, and TikTok). Systematic reports would further contribute to improving the residents’ awareness and willingness to separate waste, thereby promoting the formation of a positive atmosphere that would result in the participation of the entire community.

It is also essential to make reasonable arrangements for waste separation facilities. People can hardly undertake waste separation without the help of adequate infrastructure. Thus, measures should be taken to improve the quality of MSW separation. For example, labeling should be required on all containers used to store and transport recyclables, hazardous waste, food waste, and residual waste. It is also necessary to set up intelligent sorting centers for recyclables to improve the recycling rate of low-value items, such as glass and beverage bottles. Notably, expert supervisors should be appointed to guide urban residents during the separation and correct disposal of domestic waste.

Finally, building bidirectional incentive mechanisms of waste separation can contribute to achieving even better results. The adoption of waste separation behaviors depends on residents’ consciousness but also on the existence of appropriate incentive mechanisms that can potentially lead to the gradual internalization of separation habits as spiritual needs and to the adoption of sustainable waste separation behaviors without institutional constraints. On one hand, a new green, points-based system should be established, where an individual can gain points by collecting recyclables and then make voluntary exchange for presents. On the other hand, a waste disposal charging system should be introduced in accordance with the “polluter pays” principle, in which fees are collected based on the quantity of non-recyclable waste disposed, thereby contributing to the formation of negative incentives for residents through private cost constraints.

## 7. Conclusions

The conclusions of the present study are outlined as follows:

(1) There are direct effects of waste separation awareness on behavior. The urban residents’ cognition of waste pollution, ecological values, sense of social responsibility, perceived knowledge of waste separation, and perceived utilities of waste separation can positively influence their waste separation behaviors.
(2) Waste separation intention totally mediates the relationship between sense of social responsibility and waste separation behavior, while it partially mediates the relationships between cognition of waste pollution, ecological value, perceived utility, and waste separation behavior. Waste separation intention had no mediating effect on the relationship between perceived knowledge of waste separation and waste separation behavior.
(3) The relationship between waste separation intention and behavior can be moderated by situational factors. The cost of waste separation weakens the effect of waste separation intention on behavior, while waste separation facilities, social norms, and the publicity for China’s 14th National Games strengthen it.

## Figures and Tables

**Table 1:** Demographic distribution of 1008 respondents in Shaanxi province.

Variable	Classification	N	P	Variable	Classification (CNY)	N	P
Gender	Male	506	50.20%	Annual individual income	Less than 30,000	337	33.43%
	Female	502	49.80%		30,000–60,000	252	25.00%
Age	Younger than 21 years old	154	15.28%		60,000–100,000	294	29.17%
	21–30 years old	181	17.96%		100,000–200,000	117	11.61%
	31–40 years old	203	20.14%		200,000–500,000	6	0.59%
	41–50 years old	185	18.35%		More than 500,000	2	0.20%
	51–60 years old	166	16.47%	Annual household income	Less than 30,000	6	0.60%
	Older than 60 years old	119	11.80%		30,000–60,000	55	5.46%
Education level	Primary or middle school	350	34.72%		60,000–100,000	326	32.34%
	High school	305	30.26%		100,000–200,000	480	47.62%
	College degree	240	23.81%		200,000–500,000	120	11.90%
	Bachelor’s degree	98	9.72%		More than 500,000	21	2.08%
	Master’s or doctorate degree	15	1.49%				

Note: The total number of valid observations is 1008. “N” and “P” indicate the number and proportion of observations, respectively.

**Table 2:** Descriptive statistics of the main variables.

Variable	WSB	WSI	CWP	EV	SSR	PKWS	PUWS	CWS	WSF	SN	PNG
Mean	3.67	4.00	3.72	3.38	3.51	3.81	3.20	2.95	3.36	3.23	4.01
Standard deviation	0.72	0.78	0.84	0.88	1.02	0.75	0.99	0.62	0.80	0.89	0.61

Note: The total number of valid observations is 1008. Abbreviations: WSB, waste separation behavior; WSI, waste separation intention; CWP, cognition of waste pollution; EV, ecological value; SSR, sense of social responsibility; PKWS, perceived knowledge of waste separation; PUWS, perceived utility of waste separation; CWS, cost of waste separation; WSF, waste separation facilities; SN, social norms; PNG, publicity for China’s 14th National Games.

**Table 3:** Regression analysis results of the effects of waste separation awareness on behavior.

Variable	Standardized Coefficient	T	Sig.
CWP	0.209 ***	7.963	0.000
EV	0.272 ***	8.967	0.000
SSR	0.099 ***	3.879	0.000
PKWS	0.203 ***	7.380	0.000
PUWS	0.193 ***	6.700	0.000
R^2^	0.710		
Adj. R^2^	0.709		
F	491.495 ***		

Note: *** represent the levels of significance at 0.01. Abbreviations: WSB, waste separation behavior; CWP, cognition of waste pollution; EV, ecological value; SSR, sense of social responsibility; PKWS, perceived knowledge of waste separation; PUWS, perceived utility of waste separation.

**Table 4:** Results of the test on the mediating effect of waste separation intention.

Path	*c_1_*	*a*	*b*	*c_2_*	Is There a Mediating Effect?	Proportion of Mediating Effect
CWP → WSI → WSB	0.209 ***	0.315 ***	0.234 ***	0.135 ***	Yes	35.27%
EV → WSI → WSB	0.272 ***	0.201 ***	0.234 ***	0.225 ***	Yes	17.29%
SSR → WSI → WSB	0.099 ***	0.266 ***	0.234 ***	0.037	Yes	/
PKWS → WSI → WSB	0.203 ***	0.029	0.234 ***	0.196 ***	No	/
PUWS → WSI → WSB	0.193 ***	0.136 ***	0.234 ***	0.161 ***	Yes	16.49%

Note: *** represent the levels of significance at 0.01. Abbreviations: WSB, waste separation behavior; WSI, waste separation intention; CWP, cognition of waste pollution; EV, ecological value; SSR, sense of social responsibility; PKWS, perceived knowledge of waste separation; PUWS, perceived utility of waste separation.

**Table 5:** Results of the test on the moderating effect of waste separation cost.

Variable	Model 1		Model 2		Model 3	
	B	S.E.	B	S.E.	B	S.E.
Constant	3.675 ***	0.015	3.675 ***	0.015	3.657 ***	0.015
WSI	0.695 ***	0.019	0.675 ***	0.020	0.678 ***	0.020
CWS			−0.097 ***	0.025	−0.079 ***	0.025
WSI × CWS					−0.137 ***	0.030
R^2^	0.566		0.573		0.581	
Adj. R^2^	0.566		0.572		0.580	
F	1312.438 ***		672.944 ***		464.686 ***	

Note: *** represent the levels of significance at 0.01. B indicates unstandardized coefficient, and S.E. indicates standard error. Abbreviations: WSI, waste separation intention; CWS, cost of waste separation.

**Table 6:** Results of the test on the moderating effect of waste separation facilities.

Variable	Model 1		Model 2		Model 3	
	B	S.E.	B	SE.	B	S. E.
Constant	3.675 ***	0.015	3.675 ***	0.012	3.662 ***	0.012
WSI	0.695 ***	0.019	0.617 ***	0.016	0.606 ***	0.015
WSF			0.367 ***	0.015	0.378 ***	0.015
WSI × WSF					0.101 ***	0.018
R^2^	0.566		0.724		0.733	
Adj. R^2^	0.566		0.723		0.732	
F	1312.438 ***		1314.977 ***		918.400 ***	

Note: *** represent the levels of significance at 0.01. B indicates unstandardized coefficient, and S.E. indicates standard error. Abbreviations: WSI, waste separation intention; WSF, waste separation facilities.

**Table 7:** Results of the test on the moderating effect of social norms.

Variable	Model 1		Model 2		Model 3	
	B	S.E.	B	S.E.	B	S.E.
Constant	3.675 ***	0.015	3.675 ***	0.014	3.661 ***	0.014
WSI	0.695 ***	0.019	0.623 ***	0.018	0.626 ***	0.018
SN			0.223 ***	0.016	0.217 ***	0.016
WSI × SN					0.072 ***	0.019
R^2^	0.566		0.635		0.640	
Adj. R^2^	0.566		0.634		0.639	
F	1312.438 ***		873.071 ***		595.044 ***	

Note: *** represent the levels of significance at 0.01. B indicates the unstandardized coefficient, and S.E. indicates standard error. Abbreviations: WSI, waste separation intention; SN, social norms.

**Table 8:** Results of the test on the moderating effect of the publicity for China’s 14th National Games.

Variable	Model 1		Model 2		Model 3	
	B	S.E.	B	S.E.	B	S.E.
Constant	3.675 ***	0.015	3.675 ***	0.015	3.641 ***	0.015
WSI	0.695 ***	0.019	0.682 ***	0.020	0.640 ***	0.020
PNG			0.062 **	0.026	0.060 **	0.025
WSI × PNG					0.266 ***	0.033
R^2^	0.566		0.569		0.595	
Adj. R^2^	0.566		0.568		0.594	
F	1312.438 ***		662.381 ***		491.607 ***	

Note: ** and *** represent the levels of significance at 0.05 and 0.01, respectively. B indicates the unstandardized coefficient, and S.E. indicates standard error. Abbreviations: WSI, waste separation intention; PNG, publicity for China’s 14th National Games.

## Data Availability

The data presented in this study are available on request from the corresponding author.
